# Pancreatic medullary carcinoma developed on a pancreatic intraductal papillary mucinous neoplasm with loss of MSH2 and MSH6 expression: a case report

**DOI:** 10.1186/s13000-021-01178-0

**Published:** 2021-12-13

**Authors:** Camille Verocq, Marie-Lucie Racu, Dominique Bafort, Gloria Butorano, Luis Perez-Casanova Garcia, Julie Navez, Marc Witterwulghe, Kieran Sheahan, Niall Swan, Jean Closset, Jean-Luc Van Laethem, Calliope Maris, Nicky D’Haene

**Affiliations:** 1 Department of Pathology, Erasme Hospital, Université Libre de Bruxelles (ULB), Brussels, Belgium; 2 Centre Universitaire Inter Régional d’Expertise en Anatomie Pathologique Hospitalière (CurePath), 6040 Charleroi, Belgium; 3 Department of Hepato-Biliary-Pancreatic Surgery, Erasme University Hospital, Université Libre de Bruxelles, Brussels, Belgium; 4 Department of Gastroenterology, CHIREC Hospitals, Delta, Brussels, Belgium; 5 Department of Pathology, UCD School of Medicine, St Vincent’s University Hospital, Dublin, Ireland; 6 Department of Gastroenterology and Digestive oncology, Erasme University Hospital, Université Libre de Bruxelles, Brussels, Belgium; 7 Hôpital Erasme, Route de Lennik, 808 Laboratoire d’anatomopathologie, hôpital de jour, 2ième étage, 1070 Bruxelles, Belgium

**Keywords:** Pancreatic medullary carcinoma, Intraductal papillary mucinous neoplasm, IPMN, Microsatellite instability, Case report

## Abstract

**Background:**

Pancreatic medullary carcinoma (PMC) is a rare pancreatic tumor, usually showing the presence of microsatellite instability, mostly MLH1 silencing, and a wild-type KRAS mutation status. We report here a PMC arising from a Pancreatic Intraductal Papillary Mucinous Neoplasm (IPMN), both having *KRAS* and *TP53* mutations.

**Case presentation:**

We report the case of a 73-year-old woman presenting with right iliac fossa pain. MRI revealed a 16 mm diameter mass in the pancreas, leading to a pancreatic duct stricture and upstream a dilatation of the distal pancreatic duct of Wirsung. A fine needle aspiration was performed, and pathology analysis revealed malignant glandular cells. The patient underwent distal pancreatectomy.

Gross examination revealed an12 mm indurated white lesion, adjacent to a cystic lesion extending into the rest of the pancreatic body.

Microscopically, the cystic area represented a mixed (gastric-type and pancreatobiliary-type) IPMN, involving the main and secondary pancreatic ducts with low-grade and high-grade dysplasia. In the periphery of this IPMN, a 14mm associated invasive carcinoma was observed, characterized by focal gland formation and by poorly differentiated cells with a syncytial appearance, associated with a dense lymphoplasmocytic and neutrophilic infiltrate.

Immunohistochemical analyses showed loss of MSH2 and MSH6 expression. Microsatellite instability was confirmed by molecular test.

Molecular analysis was performed both on the invasive carcinoma and on the high-grade dysplasia IPMN, revealing the same mutation profile with *KRAS* and *TP53* mutations.

The proposed diagnosis was mixed IPMN with associated invasive medullary carcinoma that presented loss of MSH2 and MSH6 expression.

**Conclusions:**

The present case reports for the first time, at the best of our knowledge, the coexistence of IPMN lesions and PMC, both having the same molecular alterations. It also describes the second case of PMC with microsatellite instability, MSH2 and MSH6 silenced.

**Supplementary Information:**

The online version contains supplementary material available at 10.1186/s13000-021-01178-0.

## Background

Pancreatic medullary carcinoma (PMC) is a rare pancreatic tumor with 13% five-year survival rate [1, 2] which is nevertheless a better prognosis than conventional ductal adenocarcinoma. The tumor is characterized by significant tumor infiltrating lymphocytes, poorly differentiated epithelial cells with nest-like architecture, syncytial growth and pushing borders, the presence of microsatellite instability, usually with MLH1 silencing, and a wild-type KRAS mutation status [1–10]. Some poorly differentiated adenocarcinomas with EBV infection may histologically mimic medullary carcinoma [3, 4]. The present case reports for the first time, at the best of our knowledge, a PMC arising from a Pancreatic Intraductal Papillary Mucinous Neoplasm (IPMN), both having the same molecular alterations. It also describes the second case of PMC with microsatellite instability with MSH2 and MSH6 silencing, the first having been described by Banville et al. in 2006 [5].

## Case presentation

**Fig. 1:**
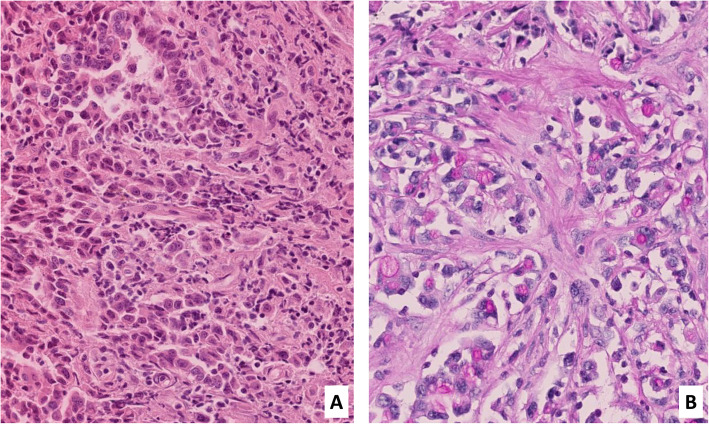
Cytopathological analysis. **A** Cytopathological analysis showing malignant cells with focal gland formation (cell block - Hematoxylin-Eosin (HE) x20). **B** The PAS-Diastase coloration confirmed the presence of mucin in the malignant cells (x20)

Herein we report the case of a 73-year-old woman, with no personal or family cancer history, presenting with right iliac fossa pain radiating into the right lumbar region since six months, without any changes in intestinal transit and with a slight increase in C-Reactive Protein (6.9 mg/L). Abdominal echography and CT Scan performed showed a multi-cystic lesion of 5 × 2 cm at the body-tail junction of the pancreas, consisting of supracentimetric cysts. Gastroduodenoendoscopy revealed grade A esophagitis and a superficial bulbar ulcer. MRI revealed a 12 mm isthmus nodular mass leading to a pancreatic duct stricture and upstream dilatation of the distal pancreatic duct of Wirsung. The blood tumor markers CA19-9 and CEA were both normal. Endoscopic Ultrasound-Guided Fine Needle Aspiration (19G) was performed and confirmed the presence of a 16 mm diameter mass on the left side of the head of the pancreas, graded uT1N0. The cytopathology analysis revealed malignant glandular cells. The PAS-Diastase confirmed the mucosecretion (Fig. 1). Immunohistochemistry revealed diffuse expression of MLH1 and PMS2 and a heterogeneous expression of MSH2 and MSH6. G12D mutation of the KRAS gene was detected using Next-Generation Sequencing.

A PET-Scan was performed, revealing hypermetabolic nodular formation of the cephalo-corporeal junction, without any other positive site. An angioscan was performed on the course, showing contact between the tumor nodule and the anterior surface of the splenomesenteric confluence, less than 180°, and with a 13 mm length. The anterior mesenteric artery and the celiac trunk were at a distance. No secondary lesion was found.

Following this assessment, the patient underwent distal pancreatectomy without splenectomy, but with cholecystectomy and splenic vessel resection. The patient has well recovered after the surgery and went home within 15 days.

**Fig. 2:**
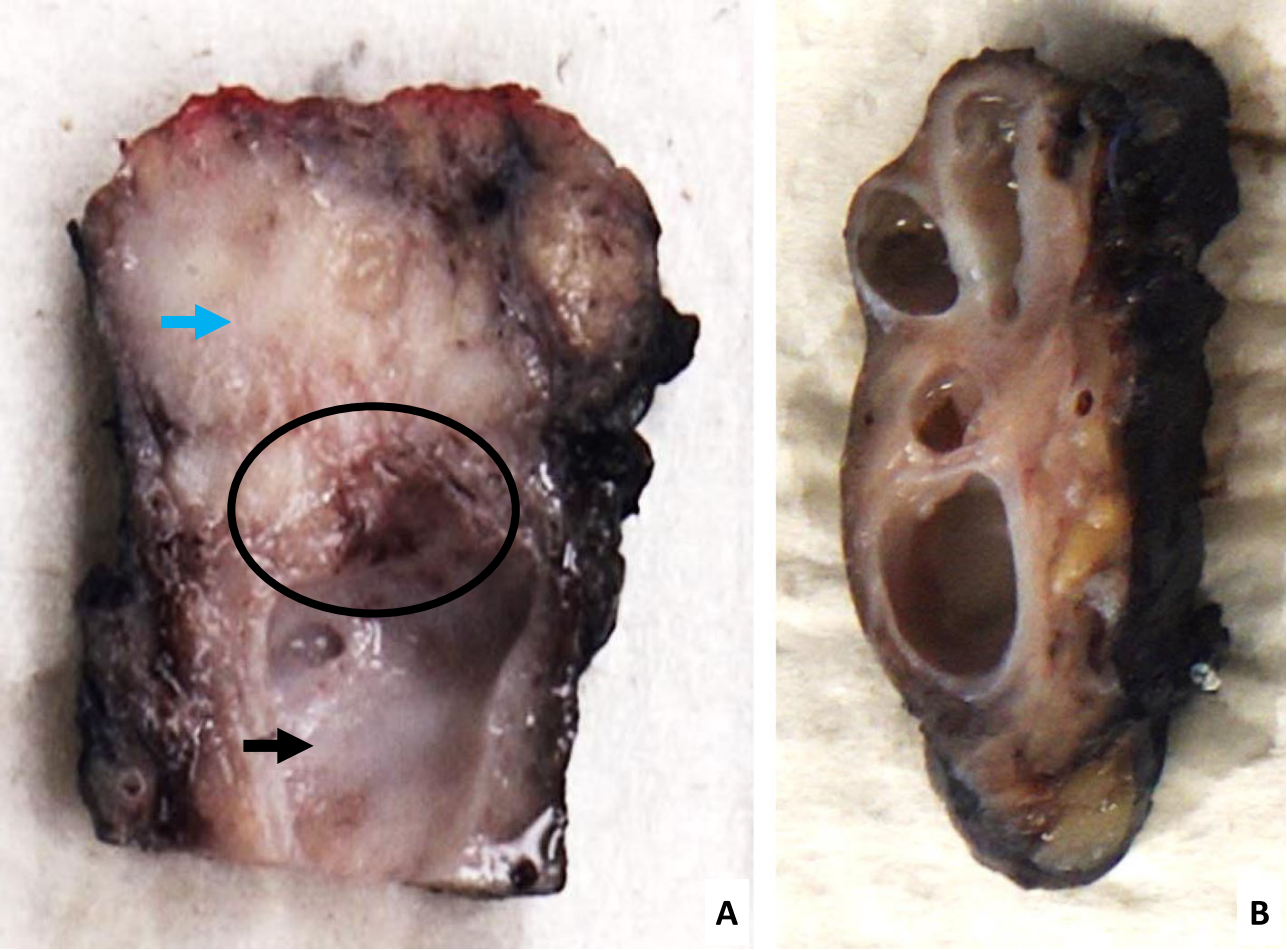
Macroscopic examination. **A** Slice of the pancreatectomy, with the section margin at the top of the image, and the body towards the bottom of the image. Blue arrow: a 12 mm indurated white lesion, close to the surgical margin. Black arrow: a 15 mm cystic lesion extending into the rest of the pancreatic body, right next to the white lesion. Circle: A contact between this whitish lesion and the cystic lesion is presumed. **B** Prolongation of cystic lesions in the rest of the pancreatic body

The gross examination of the surgical specimen revealed a left pancreatectomy, measured 10.5 × 2.5 × 1 cm. The margin section was analyzed on frozen section and there was no tumor invasion. The pancreatic section revealed a 12 mm indurated white lesion, close to the surgical margin, adjacent to a 15 mm cystic lesion extending into the rest of the pancreatic body (Fig. 2). A contact between this whitish lesion and the cystic lesion was presumed.

**Fig. 3:**
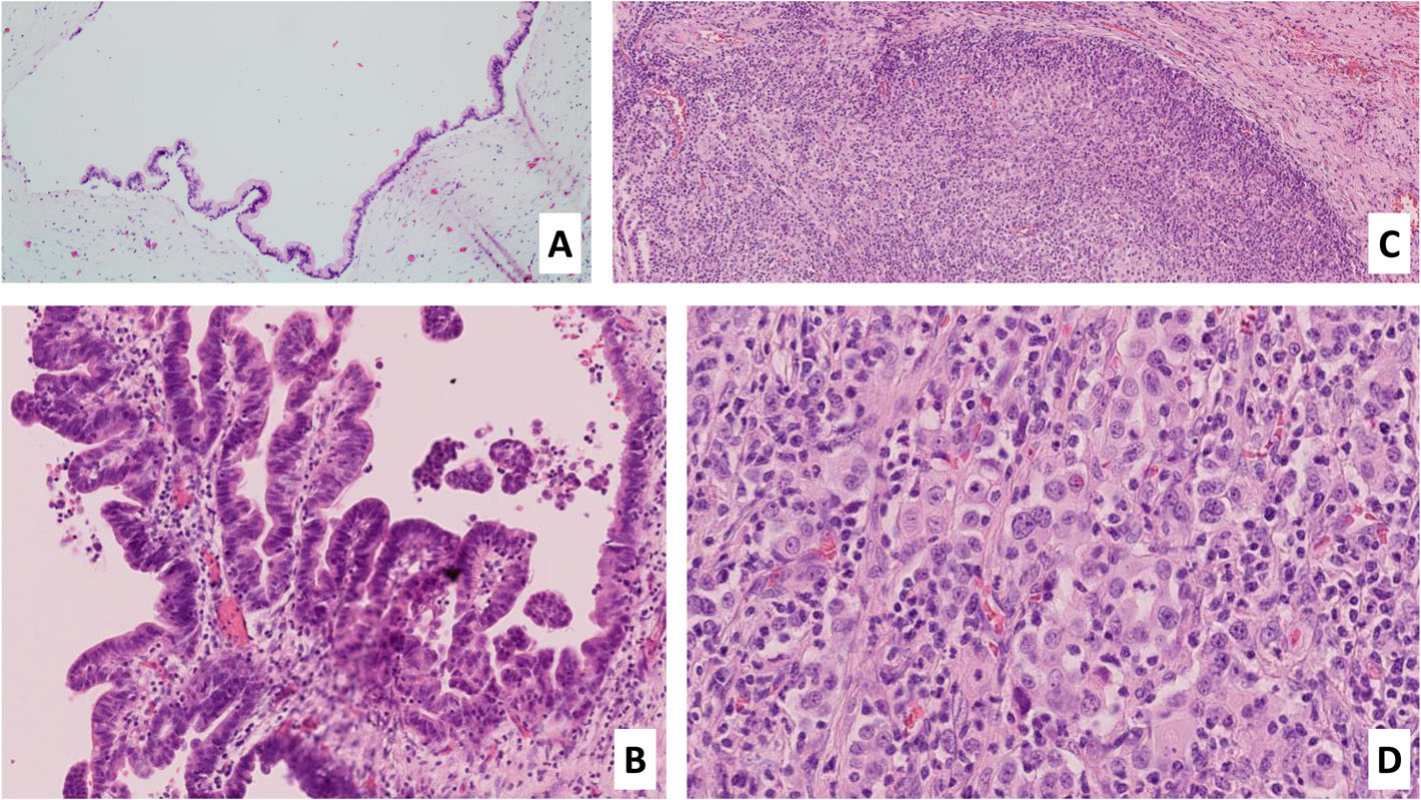
Microscopic examination of the Intraductal Pancreatic Mucinous Neoplasm (IPMN) and the Pancreatic Medullary Carcinoma (PMC). **A**: Gastric-type IPMN in low grade dysplasia (HE x5). **B**: Pancreatobiliary-type IPMN in high-grade dysplasia (HE x10). **C**: The pushing borders appearance of the PMC (HE x5). **D**: Sheets of poorly differentiated epithelial cells of the PMC, with a large nucleus and visible nucleoli; accompanied by a diffuse inflammatory infiltrate (HE x20)

**Fig. 4:**
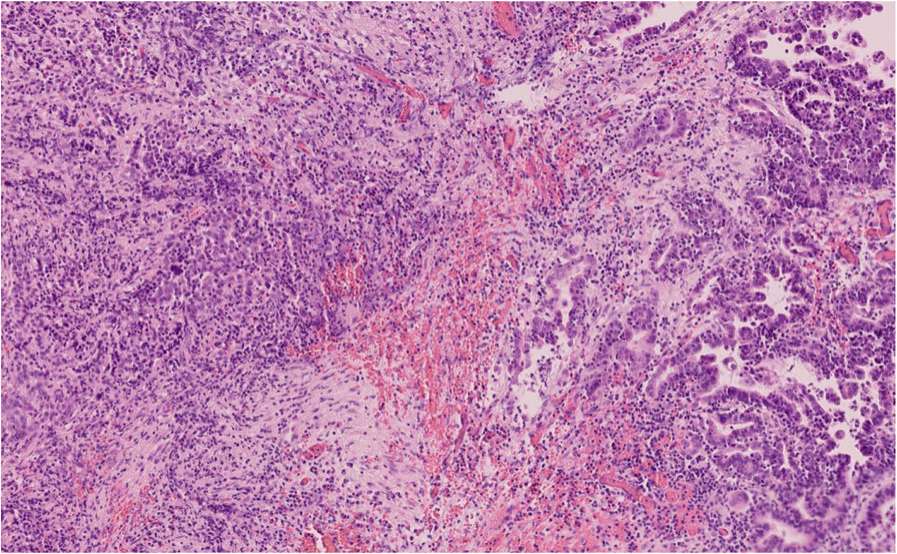
Transition between Intraductal Pancreatic Mucinous Neoplasm (IPMN) and Pancreatic Medullary Carcinoma (PMC). From right to left, the transition between pancreatobiliary-type IPMN in high-grade dysplasia, infiltrative glands and poorly differentiated epithelial cells of the invasive cancer (HE x20)

Microscopically, the cystic area represented a IPMN of mixed gastric-type and pancreatobiliary-type, involving the main pancreatic duct and secondary ducts with low-grade dysplasia for both type and high-grade dysplasia for the pancreatobiliary-type (Fig. 3). In the periphery of this IPMN, a 14 mm of long-axis associated invasive carcinoma was observed (microscopic assessment of the tumor size). This associated carcinoma was characterized by focal gland formation and by poorly differentiated cells with a syncytial appearance, associated with a dense lymphoplasmocytic and neutrophilic infiltrate (Fig. 3). The PAS special staining revealed focal mucosecretion. This histology is compatible with a PMC. This carcinoma seemed to derive from the high-grade pancreatobiliary-type IPMN (Fig. 4). No perineural or lymphovascular invasion were observed. The rest of the pancreatic parenchyma presented signs of chronic pancreatitis. No lymph node metastasis was identified.

**Fig. 5:**
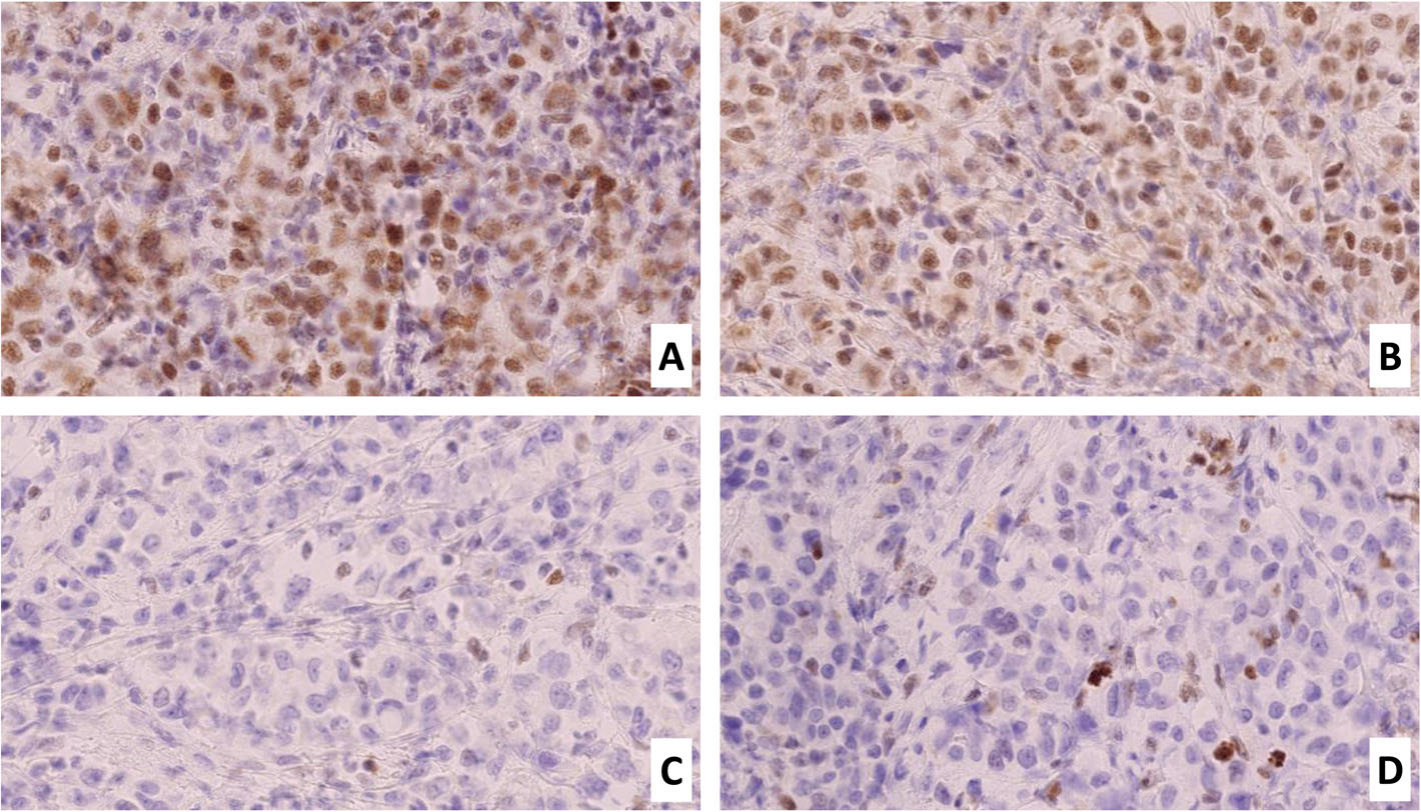
Mismatch repair protein immunohistochemistry. **A** Diffuse nuclear expression of MLH1 (x20). **B** Diffuse nuclear expression of PMS2 (x20). **C** Loss of MSH2 expression, with positive internal control (x20). **D** Loss of MSH6 expression, with positive internal control (x20).

The immunohistochemical profile of the malignant cells of the PMC was: CK7, EMA, CK19 positive, MUC5 and MUC6 focally positive, CK20, CDX2, CEH, MUC2, p53, PanTRK, chromogranin and synaptophysin negative. A focal loss of SMAD4 expression was observed. Moreover, there was a loss of MSH2 and MSH6 expression, both in the IPMN and in the PMC, suggesting microsatellite instability (Fig. 5). This was confirmed by Polymerase Chain Reaction (Idylla assay). A genetic counselling has been requested. The tumor was staged as: pT1cN0 according to UICC 2017 [6].

Molecular analysis using targeted Next Generation Sequencing was performed as previously described [7] (50 genes panel, Cancer Hotspot Panel, Ampliseq, Life technologies) both on the invasive carcinoma and on the high-grade dysplasia IPMN revealing the same mutation profile: *KRAS* G12D and *TP53* S90Pfs*33 mutations.

Because Epstein-Barr Virus (EBV) infection can mimic medullary carcinoma, EBV Chromogenic In Situ Hybridization was performed but was negative [3, 4].

The proposed diagnosis was mixed IPMN with associated invasive medullary carcinoma that presented loss of MSH2 and MSH6 expression.

## Discussion and conclusions

In the 5th edition of the WHO classification of the digestive system tumors [3], PMC is reported as a histologic subtype of pancreatic ductal adenocarcinoma. Although this carcinoma is of ductal lineage, it is characterized by distinct prognostic and molecular features. Regarding prognosis, despite a five-year survival rate estimated at 13% [1, 2], PMC still seems to have a better prognosis than conventional ductal pancreatic adenocarcinoma [3]. Regarding molecular features, PMC are often characterized by a microsatellite instability and a wild-type KRAS status. Moreover, identification of a PMC should trigger an investigation for Lynch syndrome [1–5, 8–11].

PMC is histologically characterized by nests of poorly differentiated epithelial cells, which do not form glands, by syncytial growth, and a large number of tumor infiltrating lymphocytes. The tumor site is well delineated and pushes the adjacent fibrous stroma rather than infiltrating it (“pushing borders”) [1–3, 5, 8–10]. All these characteristics were present in our patient. As soon as a lymphocyte-rich stromal and intra-tumoral infiltrate are found in a poorly differentiated pancreatic carcinoma, a EBV Chomogenic In Situ Hybridization should be performed, to exclude poorly differenciated carcinoma with EBV infection, which can mimic a medullary carcinoma [3, 4]. In our case, this analysis was negative.

PMC must be distinguished from pancreatic ductal adenocarcinoma, EBV-related poorly differentiated adenocarcinoma and neuroendocrine neoplasms [11]. Pushing borders and syncytial pattern instead of infiltrative borders are more in favor of PMC than ductal adenocarcinoma. EBV ISH allows to differentiate PMC from EBV-related poorly differentiated adenocarcinoma, and neuroendocrine IHC, PMC from neuroendocrine carcinomas. Neuroendocrine immunostainings (chromogranin and synaptophysin) were negative in the present case.

We herein describe the second case of PMC associated with microsatellite instability MSH2 and MSH6 silenced. So far, it is established in the literature that PMC are associated with microsatellite instability and occur either sporadically or as part of Lynch syndrome [1–5, 8–11]; and most often, the affected gene/protein is MLH1 [4, 5]. In addition, some authors have shown that poorly differentiated pancreatic carcinomas were associated with microsatellite instability [1, 5, 8, 12], and therefore, the diagnosis of medullary carcinoma should be investigated, as well as the possibility of a hereditary syndrome [8, 10, 11].

The first case of PMC with microsatellite instability of MSH2 and MSH6 was described by Banville et al. in 2006 [5]. The patient, who was 63 years old at the time of the discovery of his pancreatic carcinoma, had developed two adenocarcinomas (rectal and caecal) a few years earlier. All three tumors had lost the expression of MSH2 and MSH6. A Lynch syndrome had been detected with MSH2 gene germline mutation. Currently, we have no additional information on the genetic profile of our patient, therefore not allowing us to assert the presence of a Lynch syndrome. Nevertheless, her pancreatic cancer is the first cancer she developed.

The etiology and pathogenesis of PMC are not well described. However, in the study of Wilentz et al., a medullary phenotype was significantly associated with a family history of any cancer in a first-degree relative, MSI, and a wild-type KRAS gene [4]. Moreover, recently, Kryklyva et al. reported the first case of PMC associated with a POLE mutation [13]. These authors highlighted that both MSI and POLE-mutated cancers are usually associated with high tumor mutational burden, resulting in a prominent antitumor immune response and overall better prognosis [13].

At the best of our knowledge, our case is also the first in the literature to highlight the development of PMC from an IPMN lesion. IPMN is an intraepithelial neoplasia consisting of mucin-producing columnar cells, developing in the main pancreatic duct or its branches, most often in the pancreatic head [2, 3]. There are three types of IPMN: intestinal-type, gastric-type and pancreatobiliary-type. Usually, the first is a precursor of colloid pancreatic carcinoma, and the other two of tubular pancreatic carcinoma [3, 9]. In the present case, we find these last two phenotypes within the IPMN, but a medullary pattern in the invasive part.

Concerning the molecular analysis, performed on the IPMN in high-grade dysplasia and on the medullary invasive part, we identified a G12D mutation of the KRAS gene and a mutation of the TP53 gene (s90Pfs*33). This molecular profile is quite consistent with the mutations generally found in IPMN and tubular carcinoma lesions. Indeed, 60 to 80% of IPMN show a KRAS mutation, as well as an overexpression of TP53 in 10 to 40% of high-grade IPMN, and 40 to 60% of invasive carcinomas developed on IPMN [3]. In contrast, medullary carcinoma is associated with a wild-type KRAS gene [2–5, 8, 12]. Nevertheless, Wilentz et al. showed that medullary carcinomas also present mutations of codon 12 of the KRAS gene in four out of 13 PMC cases [4].

Our case of PMC is associated with IPMN lesions, although the latter is mostly seen with ductal carcinoma [2, 3, 9]. However, given the same molecular profile of IPMN and PMC, and the microscopic association of the two lesions, the question of a relationship may be asked, which has never yet been the case in the literature. However, in the review by Wilentz et al., they showed that 45% of patients with PMC had associated low-grade Pancreatic Intraepithelial Neoplasia (PanIN) lesions, without showing a causal connection between the two [4]. To date, the pathogenesis of IPMN is still not elucidated. Indeed, it is not known whether it develops de novo, on PanIN lesions, or whether the two mechanisms coexist [2, 3, 14].

As it is the first case describing IPMN and PMC association, however, an incidental finding cannot be excluded, and further studies are needed to evaluate this association.

In conclusion, the present case reports for the first time, at the best of our knowledge, the coexistence of IPMN lesions and PMC, both having the same molecular alterations. It also describes the second case of PMC with microsatellite instability characterized by MSH2 and MSH6 silencing, the first having been described by Banville et al. in 2006 [5].

## Supplementary information

**Additional file 1**

## Data Availability

Not applicable.

## Abbreviations

EBV: Epstein-Barr Virus
HE: Hematoxylin-Eosin
IPMN: Intraductal Papillary Mucinous Neoplasm
PanIN: Pancreatic Intraepithelial Neoplasia
PMC: Pancreatic Medullary Carcinoma
