# Supplementary material for: Pancreatic medullary carcinoma developed on a pancreatic intraductal papillary mucinous neoplasm with loss of MSH2 and MSH6 expression: a case report
Source: Diagn Pathol. 2021 Dec 13;16:117. doi: 10.1186/s13000-021-01178-0 (PMC8667442; doi:10.1186/s13000-021-01178-0)
Supplement: Supplementary file 1 — Additional file 1 [file 13000_2021_1178_MOESM1_ESM.pdf]

CARE
case report guidelines

CARE Checklist of information to include when writing a case report

(cc) BY-NC-ND

Topic	Item	Checklist item description	Reported on Line
Title	1	The diagnosis or intervention of primary focus followed by the words "case report"	Page 1 Line 1
Key Words	2	2 to 5 key words that identify diagnoses or interventions in this case report, including "case report"	Page 3 Line 68
Abstract (no references)	3a	Introduction: What is unique about this case and what does it add to the scientific literature?	Page 2 Line 36
	3b	Main symptoms and/or important clinical findings	Page 2 Line 41
	3c	The main diagnoses, therapeutic interventions, and outcomes	Page 2 Line 41
	3d	Conclusion—What is the main "take-away" lesson(s) from this case?	Page 2 Line 63
Introduction	4	One or two paragraphs summarizing why this case is unique (**may include references**)	Page 3 Line 72
Patient Information	5a	De-identified patient specific information.	Page 3 Line 88
	5b	Primary concerns and symptoms of the patient.	Page 3 Line 88
	5c	Medical, family, and psycho-social history including relevant genetic information	Page 3 Line 88
	5d	Relevant past interventions with outcomes	Page 3 Line 88
Clinical Findings	6	Describe significant physical examination (PE) and important clinical findings.	Page 3 Line 88
Timeline	7	Historical and current information from this episode of care organized as a timeline	Not Applicable
Diagnostic Assessment	8a	Diagnostic testing (such as PE, laboratory testing, imaging, surveys).	Page 3 Line 93
	8b	Diagnostic challenges (such as access to testing, financial, or cultural)	Not Applicable
	8c	Diagnosis (including other diagnoses considered)	Page 6 Line 157
	8d	Prognosis (such as staging in oncology) where applicable	Page 5 Line 145
Therapeutic Intervention	9a	Types of therapeutic intervention (such as pharmacologic, surgical, preventive, self-care)	Page 4 Line 113
	9b	Administration of therapeutic intervention (such as dosage, strength, duration)	Not Applicable
	9c	Changes in therapeutic intervention (with rationale)	Not Applicable
Follow-up and Outcomes	10a	Clinician and patient-assessed outcomes (if available)	Not Applicable
	10b	Important follow-up diagnostic and other test results	Not Applicable
	10c	Intervention adherence and tolerability (How was this assessed?)	Not Applicable
	10d	Adverse and unanticipated events	Not Applicable
Discussion	11a	A scientific discussion of the strengths AND limitations associated with this case report	Page 6 Line 160
	11b	Discussion of the relevant medical literature **with references**.	Page 6 Line 160
	11c	The scientific rationale for any conclusions (including assessment of possible causes)	Page 6 Line 160
	11d	The primary "take-away" lessons of this case report (without references) in a one paragraph conclusion	Page 8 Line 227
Patient Perspective	12	The patient should share their perspective in one to two paragraphs on the treatment(s) they received	Not Applicable
Informed Consent	13	Did the patient give informed consent? Please provide if requested	Yes ☐ No ☑
